# High-Fat Diet Increased Oxidative Stress and Mitochondrial Dysfunction Induced by Renal Ischemia-Reperfusion Injury in Rat

**DOI:** 10.3389/fphys.2021.715693

**Published:** 2021-09-03

**Authors:** Priyanka N. Prem, Gino A. Kurian

**Affiliations:** ^1^School of Chemical and Biotechnology, SASTRA Deemed University, Thanjavur, India; ^2^Vascular Biology Lab, SASTRA Deemed University, Thanjavur, India

**Keywords:** ischemia-reperfusion injury, high fat diet, renal injury, mitochondria, oxidative stress

## Abstract

Renal ischemia-reperfusion (IR) injury is one of the major causes of acute kidney injury influenced by the ischemic duration and the presence of comorbidities. Studies have reported that high-fat diet consumption can induce renal lipotoxicity and metabolic dyshomeostasis that can compromise the vital functions of kidney. This study aimed to evaluate the impact of a high-fat diet in the recovery of renal tissue from IR and explored the cellular pathology. In this study, 24 male Wistar rats were divided into two groups: normal diet (ND; *n* = 12) and high-fat diet (HD; *n* = 12), which were further subdivided into sham and IR groups at the end of the dietary regimen. The high-fat diet was introduced in 4-week-old rats and continued for 16 weeks. IR was induced by bilateral clamping of the renal peduncle for 45 min, followed by 24 h of reperfusion. Blood chemistry, estimated glomerular filtration rate (eGFR), mitochondrial function, and oxidative stress analysis were carried out to study the pathological changes. The rats fed with HD showed a decreased eGFR and elevated plasma creatinine, thereby compromised kidney function. Subcellular level changes in HD rats are deceased mitochondrial copy number, low PGC-1α gene expression, and declined electron transport chain (ETC) enzymes and adenosine triphosphate (ATP) level. Upon IR induction, HD rats exhibited severely impaired renal function (eGFR-0.09 ml/min) and elevated injury markers compared with ND rats. A histological analysis displayed increased tubular necrosis and cast formation in HD-IR in comparison to ND-IR. The oxidative stress and mitochondrial dysfunction were more prominent in HD-IR. *In vitro* protein translation assessment revealed impaired translational capacity in HD-IR mitochondria, which suggests mitochondrial changes with diet that may adversely affect the outcome of IR injury. High-fat diet consumption alters the normal renal function by modifying the cellular mitochondria. The renal changes compromise the ability of the kidney to recover from ischemia during reperfusion.

## Introduction

Diet, specifically designed for the kidney, can be promote its health and slow down its progression to failure (Rysz et al., 2017). Previous studies have reported that dietary restriction of protein, fat, salt, and phosphate can preserve kidney function (Huang et al., 2013; Ko et al., 2017; Elder et al., 2018). Diet rich in high fat is common in many countries and can contribute to the development and progression of non-communicable diseases, such as cardiovascular diseases, Alzheimer's disease, and acute and chronic kidney injury (World Health Organization, 2010). Epidemiological studies support an increased prevalence of acute renal disease in patients consuming a large amount of high-fat diet (HD) (Rysz et al., 2017). HD is reported to induce glomerulopathy and proximal convoluted tubule injury in kidney (Szeto et al., 2016). In addition, it can impact lysosomal dysfunction and impaired autophagy (Yamamoto et al., 2017). Long-term consumption of a HD affects the energy balance of the renal cell and leads to the accumulation of lipids, which can cause renal mitochondrial dysfunction (Muller et al., 2019). Impaired mitochondrial biogenesis, altered mitochondrial dynamics, and declined renal mitochondrial respiration are associated with high-fat consumption in heart (Chen D. et al., 2018). Kidney, being an energetically active organ, is expected to have similar changes as that of the heart (Sun et al., 2020). Nephrons are rich in mitochondria, thus HD-linked renal tubular cell injury and alterations in glomeruli, fibrosis, and podocyte damage that can promote the transition of acute to chronic kidney injury may be associated with mitochondrial function (Sun et al., 2020).

Ischemia-reperfusion injury (IR) is an unavoidable injury associated with different surgical interventions, such as kidney transplantations, partial nephrectomy, cardiac bypass surgery, and other medical conditions linked to hypotension and sepsis (Ponticelli, 2014; Malek and Nematbakhsh, 2015). The major pathophysiological mediators associated with renal IR are inflammatory molecules and reactive oxygen species (ROS) release as the secondary effect of bioenergetic imbalance induced by ischemia (Malek and Nematbakhsh, 2015). Mitochondria known to control the redox balance of the cell that regulate the bioenergetics and thus the dysfunction of mitochondria resulted from IR is considered to be another critical player in the pathology (Martin et al., 2019). Decline in the removal of damaged mitochondria associated with renal IR promotes the kidney injury and indicates impaired mitophagy. Similarly, many studies have noted declined mitochondrial function and increased mitochondrial fusion linked to renal IR (Wang et al., 2020a,b; Zhang et al., 2021).

Renal IR-induced acute kidney damage contributes to high morbidity and mortality, where the effective strategy to prevent the injury in the clinic is a challenge (Han and Lee, 2019). The dietary influence of IR is reported to have conflicting results in different organs challenged to revascularization procedures. For example, a decreased myocardial tolerance to IR damage was observed in the studies using hyperphagia-induced obese insulin-resistant male rats (Webster et al., 2017). In contrast to the finding, relatively long-term administration of the two obesity-inducing diets—high carbohydrate and HDs resulted in cardioprotection against IR-induced damage (Salie et al., 2014). Despite the vast amount of research studies carried out to assess the susceptibility of the heart to IR damage in HD-induced animal models, relatively little is known about the effects of HD on kidney IR *per se* in this scenario.

To address this key deficit in our knowledge, this study aimed to investigate the effects of HD intake on the morphology and renal function of adult rats. Further, we evaluate the impact of HD on renal recovery from IR and will explore the underlying mechanism involved in the pathology.

## Materials and Methods

### Animals

All experiments were carried out with male Wistar rats (180–200 g) procured from the central animal facility, SASTRA Deemed University, Tamil Nadu, India. The rats were maintained on a 12 h light/dark cycle under controlled temperature conditions (25 ± 2°C) with access to food (1328, Altromin Gmbh, Germany) and water. The animal experiments were conducted by the guidelines of the Committee for the Purpose of Conduct and Supervision of Experiments on Animals, India, with prior approval from the Institutional Animal Ethical Committee (613/SASTRA/IAEC/RPP).

### High-Fat Diet Preparation

Maintenance diet based on cereal (soy, wheat, and corn) fixed formula, which is free of alfalfa and fish/animal meal and deficient in nitrosamines, was used for the preparation of high-fat diet (HD) by the addition of 40% fat (beef tallow) to the powdered chow pellets and then re-pelleted using hand pellet presser. The maintenance semi-purified diet was procured from Altromin (Spezialflutter GmbH & Co., Germany; prepared according to the AIN-93G formulation). The total energy of the standard diet for normal animals was 395 kcal/100 g (65% carbohydrates, 24% proteins, and 11% fat) and for the high fat diet has total energy of 540 kcal/100 g (40% carbohydrates, 20% proteins, and 40% fat).

### Study Design

The rats were assigned to two major groups (*n* = 12 per group), namely normal diet-fed (ND) and high-fat diet-fed (HD). The HD group rats were maintained on a HD for 16 weeks, whereas the ND group rats were maintained on a ND. Body weight was recorded at every 2-week interval. At the end of 16 weeks, each group was subdivided into two subgroups (*n* = 6)—(1) sham—in this group, right and left renal pedicles of rats were surgically exposed and kept for 45 min without clamping; (2) IR—the renal peduncle of the rats were exposed and ligated to induce ischemia for 45 min and removed the ligation to initiate the reperfusion for 24 h. At the end of the study, the rats were euthanized using an anesthetic overdose of isoflurane and collected kidneys for biochemical and morphological assessment. From each group, kidneys (*n* = 3) were stored in formalin for histopathological analysis using hematoxylin/eosin.

### Blood Analysis

The blood samples were collected in tripotassium ethylenediaminetetraacetic acid (K3EDTA) tubes from all rats before the surgery and on the day of necropsy. Plasma was separated by centrifuging the tubes at 3,000 rpm for 10 min and stored at −80°C for further analysis.

The plasma was used for the estimation of kidney injury markers (urea and creatinine), lipid profile (LDL-C, HDL-C, total cholesterol, and triglycerides), liver function test (SGOT and SGPT), and glucose, whereas urine samples were used for the analysis of blood urea nitrogen (BUN) and albumin estimation using kit (Agape, India) method according to the instructions from the manufacturer. All spectrophotometric measurements were carried out using the Synergy H1 multimode reader (BioTek, USA).

### Glomerular Filtration Rate

Animals used for urine collection were housed in metabolic cages. The animals had free access to water during the entire experiment, which could last up to 24 h post-surgery. The urine was collected at 4, 8, and 24 h after the surgery, and the total urine volume was determined. Creatinine and BUN were measured in both plasma and urine.

Estimated glomerular filtration rate (eGFR) calculation was carried out using the following equation (Pestel et al., 2007):

- Creatinine clearance = (1000 * urine volume * concentration of creatinine in urine)/concentration of creatinine in serum
- BUN clearance = (urine volume * concentration of BUN in urine)/concentration of BUN in serum
- eGFR = mean of (creatinine clearance, BUN clearance).

### Mitochondrial Analysis

The differential centrifugation technique was used to isolate rat kidney mitochondria as described elsewhere (Afanasyeva et al., 2018). After estimation of protein with Lowry's method and normalization, mitochondrial electron transport chain (ETC) enzyme activities were measured spectrophotometrically by using specific donor-acceptor oxidoreductase activities in 0.1 M phosphate buffer. Rotenone-sensitive NADH oxidoreductase was used to assess the complexes I activity; succinate decylubiquinone 2,6-dichlorophenolindophenol (DCPIP) reductase assess the complex II activity, ubiquinol cytochrome-c reductase to assess complex III, and cytochrome c oxidase (Complex IV) were measured as per the protocol described previously (Ansari et al., 2019).

The ATPlite (Perkin Elmer, Melbourne) system was used for the measurement of adenosine triphosphate (ATP) levels in the isolated mitochondria of all the experimental groups. The determination was based on the production of light caused by the reaction of ATP with added luciferase and α-luciferin. ATP-producing ability was evaluated by incubating isolated mitochondria in glutamate/malate (5/2.5 mM) and succinate (2.5 mM) energized medium for about 10 min, and luminescence was read (Ansari et al., 2019).

### Estimation of Mitochondrial Copy Number and Gene Expression Level of Proliferator-Activated Receptor Gamma Coactivator 1 Alpha (PGC 1α)

The expression for the mitochondrial encoded ND1 (F-CCACCGCGGTCATACGATTA, R-AGGGCTAAGCATAGTGGGGT) and nuclear-encoded β actin (F- GTGTGGTCAGCCCTGTAGTT, R- CCTAGAAGCATTTGCGGTGC), PGC 1α (F- GAGGGACGAATACCGCAGAG, R- CTCTCAGTTCTGTCCGCGTT), PINK (F- TGTATGAAGCCACCATGCCC, R- TCTGCTCCCTTTGAGACGAC) and PARKIN (F- AGTTTGTCCACGACGCTCAA, R- CAGAAAACGAACCCACAGCC) was analyzed using qPCR (ABI7500, Thermo Scientific, USA) in rat kidney samples. ND1 expression was analyzed in cellular DNA, whereas proliferator-activated receptor gamma coactivator 1 alpha (PGC 1α), PINK, and PARKIN was estimated in mRNA and normalized with β actin in respective samples. The DNA isolation was carried out using phenol-chloroform-isoamyl alcohol method according to the instructions of the manufacturer (Himedia, Mumbai). The mRNA extraction was carried out using TRIzol reagent (15596026, Thermo Scientific, USA) as per the instructions, and gene expression was quantified using Sybr green chemistry (F415, Thermo Scientific, USA). The expression of genes was calculated as per the procedure of Livak and Schmittgen (2001).

### Oxidative Stress Assessment

The antioxidant levels in renal homogenate and isolated mitochondria, such as reduced glutathione (GSH) and superoxide dismutase (SOD), were estimated by the previously reported methods with slight modifications. Catalase activity was determined by measuring the decrease in absorbance at 240 nm due to the decomposition of H_2_O_2_ in a UV recording spectrophotometer. The lipid peroxidation product MDA level was evaluated by measuring thiobarbituric acid-reactive substances level as previously reported (Mahalakshmi and Kurian, 2018).

### *In vitro* Protein Synthesis

*In vitro* protein synthesis in isolated mitochondria performed as per the previous procedure by Fernandez-silva et al. with slight modifications (Fernandez-Silva et al., 2007). Briefly, the isolated mitochondria are suspended in MATE buffer (25 mM sucrose, 75 mM Sorbitol, 10 mM KCl, 0.05 mM Tris-HCl, and 10 mM K_2_HPO_4_-pH 7.4) containing 10 mM glutamate and 2.5 mM malate, 1 mM ADP, and 1 mg/ml of fatty acid-free BSA. To initiate the translation, 100 μg/ml emetine, 100 μg/ml cycloheximide, and 10 μM of the 20 L-amino acids were added to the medium and incubated for 25 min with gentle shaking. At the end of 25 min, the mitochondria were pelleted and suspended in lysis buffer and proceeded with sodium dodecyl sulfate–polyacrylamide gel electrophoresis (SDS PAGE).

For SDS PAGE, a 12% resolving gel was used. Following electrophoresis, gels were incubated in Coomassie blue stain for 4 h and kept in destaining solution for 2 h. Gels were imaged using Quantity One software (Bio-Rad, CA, USA), and intensity was calculated using Image J software.

### Statistical Analysis

A statistical analysis was carried out by Prism version 8 (Graph Pad Software Inc., San Diego, CA, USA). Two-way analysis of variance (ANOVA), followed by Dunnet's post-test, was used to analyze the data. The experimental results were expressed as mean ± SD, and a *P* < 0.05 was considered statistically significant.

## Results

### Effect of High-Fat Diet on Body Weight and Organ/Body Weight

**Figure 1:**
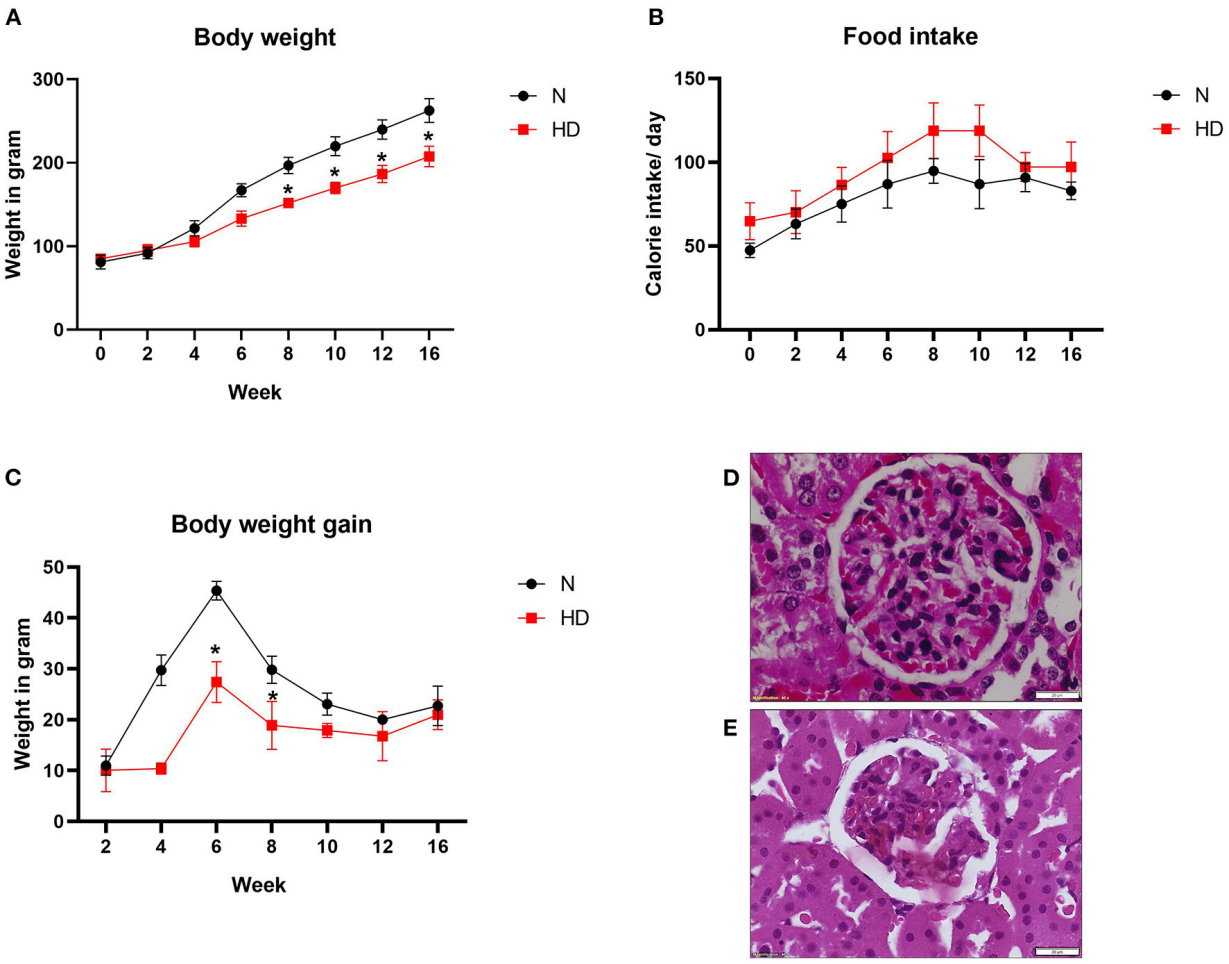
Body weight and renal pathology analysis of ND and HD rats **(A)** food intake, **(B)** body weight gain, and **(C)** body weight. *p < 0.05 vs. ND. The data are presented as mean ± SD (*n* = 6/group) **(D)** H&E stained images of kidney from ND and **(E)** HD (*n* = 3/group). The images are presented at 40x magnification. ND, Normal diet fed; FD, High fat diet fed.

To investigate the effect of HD on body weight, we kept Wistar rats on high-fat diet (HD) for 16 weeks. As shown in Figures 1A,B, the rats fed with HD showed a lower food intake than normal diet (ND) rats but in terms of caloric value, the intake by HD rats was slightly higher. Compared with ND-fed rats, body weight of HD rats was low at the end of 16 weeks of feeding (Figure 1C).

**Figure 2:**
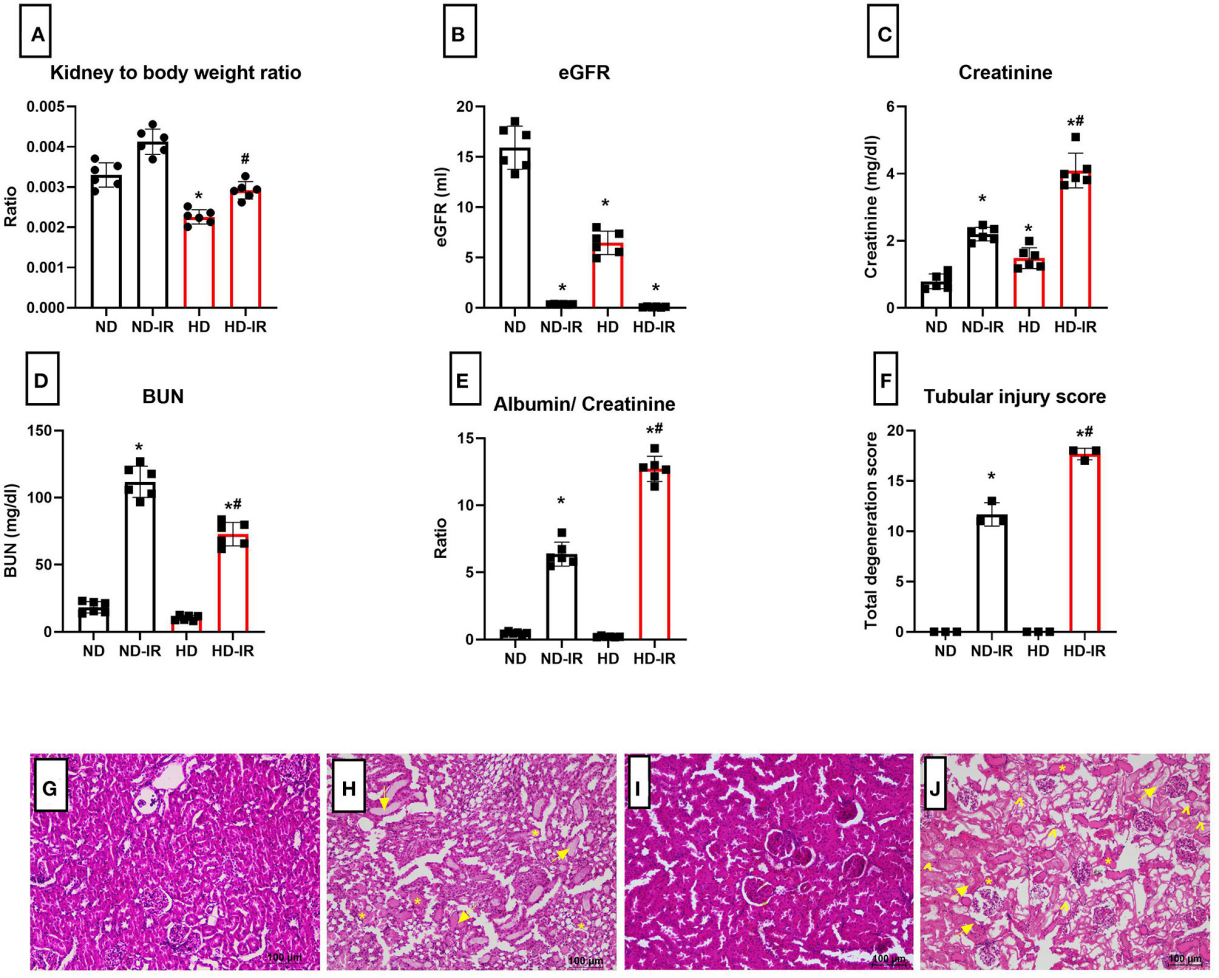
Renal physiology and biochemistry analysis of ND and HD rats subjected to IR **(A)** kidney to body weight ratio, **(B)** eGFR, **(C)** plasma creatinine, **(D)** plasma BUN, **(E)** urinary albumin/creatinine ratio, and **(F)** renal injury score from **(H,E)** staining. *p < 0.05 vs. ND, ^**#**^*p* < 0.05 vs. ND-IR. The data are presented as mean ± SD (*n* = 6/group). H&E stained images for ND and HD renal tissue subjected to IR injury **(G)** ND, **(H)** ND-IR, **(I)** HD, and **(J)** HD-IR (*n* = 3/group). The images are presented at 10x magnification. *Indicates tubular cast formation, arrow indicates extensive tubular dilatation, and arrow head indicates tubular necrosis. ND, Normal diet fed; FD, High fat diet fed; IR, Ischemia-reperfusion.

The renal histopathology showed marked difference in the glomeruli of HD rat in comparison with ND rats. Glomerular retraction, reduction in Bowman's capsule area, and Bowman's space area were observed in the HD kidney, which were absent in the ND rat kidney (Figures 1D,E). The kidney-to-body weight ratio was found to be decreased in the HD-fed rat compared with the ND-fed animal (Figure 2A).

### The Effects of High-Fat Diet on Blood Biochemistry

The effects of diets on the levels of plasma lipid profile are shown in Table 1. After 16-week treatment with HD, plasma triglycerides (HD-189.2 ± 22.7, ND-120.4 ± 19.3), total cholesterol (HD-234.3 ± 12, ND-147.5 ± 8.2), LDL-C (HD-95.8 ± 7.1, ND-47.4 ± 4.1), and HDL-C (HD-106.5 ± 4.2, ND-67.1 ± 6.4) concentrations of HD rats were significantly (*p* < 0.05) increased. The glucose level showed no difference between HD and ND rats. Plasma transaminases as markers of liver disease were evaluated and found to be significantly (*p* < 0.05) elevated in the HD group (Table 1), indicating possible liver injury, which was visually observed during the surgery.

**Table 1:** The changes in blood biochemistry and abdominal fat content of rats fed with normal diet and high fat diet.

**Parameters**	**ND (*n =* 6)**	**HD (*n =* 6)**
Total cholesterol (mg/dl)	147.5 ± 8.2	234.3 ± 12^*^
LDL-C (mg/dl)	47.4 ± 4.1	95.8 ± 7.1^*^
HDL-C (mg/dl)	67.1 ± 6.4	106.5 ± 4.2^*^
Triglycerides (mg/dl)	120.4 ± 19.3	189.2 ± 22.7^*^
Glucose (mg/dl)	154.5 ± 13.4	149.0 ± 20.5
SGPT (mg/dl)	24.0 ± 5.4	50.9 ± 6.9^*^
SGOT (mg/dl)	72.5 ± 9.7	103.1 ± 11.2^*^
Abdominal fat/body wt	0.010 ± 0.002	0.065 ± 0.004^*^

*The data are presented as mean ± SD (n = 6/group). ND, Normal diet fed; FD, High fat diet fed*.

* *p < 0.05 vs. ND*.

### Effect of High-Fat Diet on Renal Physiology and Injury Markers

In order to assess the HD impact on renal function, we analyzed the plasma markers and eGFR (Figures 2B–D). According to Figure 2B, the basal level of GFR in HD rats is significantly declined in comparison with the ND rats. The plasma creatinine level indeed showed a significant increase in HD rats, but values were in the normal range (in mg/dl ND-0.67 ± 0.02; HD-1.37 ± 0.3). Interestingly, the HD rats showed an insignificant reduction in the plasma blood urea nitrogen (BUN) and urinary albumin/creatinine ratio compared with the ND rats (Figures 2D,E).

### Effect of High-Fat Diet in the Physiological Recovery of Kidney From Ischemia-Reperfusion (IR)

Figures 2B–E shows the physiological parameters of the rats evaluated 24 h after the surgery. The mean kidney weight/body weight ratio was slightly higher in the IR group, but compared to ND, HD showed a higher weight gain (ND-IR-20%, HD-IR-30%) from its own control group (Figure 2A).

The IR-associated renal dysfunction was evidenced in the HD rats by a significant (*p* < 0.05) reduction in eGFR (in ml/day ND-IR-0.35 ± 0.02, HD-IR-0.09 ± 0.04) (Figure 2B), together with a significant (*p* < 0.05) elevation of creatinine (in mg/dl ND-IR-2.20 ± 0.2, HD-IR- 3.9 ± 0.5) in the serum (Figure 2C) and urinary albumin-to-creatinine ratio (ND-IR-6.09 ± 0.85, HD-IR-13.43 ± 0.73) (Figure 2E), compared with the ND group.

### Effect of High-Fat Diet in the Ischemia-Reperfusion (IR)-Associated Renal Tubular Injury

The histopathological analysis of the kidneys from both diet-fed groups exhibited normal renal tubular pathology (Figures 2F–J). Upon IR induction, compared to the sham kidneys, both ND-IR and HD-IR showed severe tubular degeneration with vacuolization in proximal tubules, glomerular congestion, hemorrhage, degeneration of tubular architecture, tubular dilatation, and loss of brush border and cast formation (Figures 2H,J). In comparison with ND-IR, an escalated injury found HD-IR due to severe congestion, tubular dilatation, and marked tubular cast formation (Figure 2H). In addition, severe tubular necroses were also observed in the HD-IR group.

### Effect of High-Fat Diet on the Mitochondrial Dysfunction Linked to Ischemia-Reperfusion (IR)

**Figure 3:**
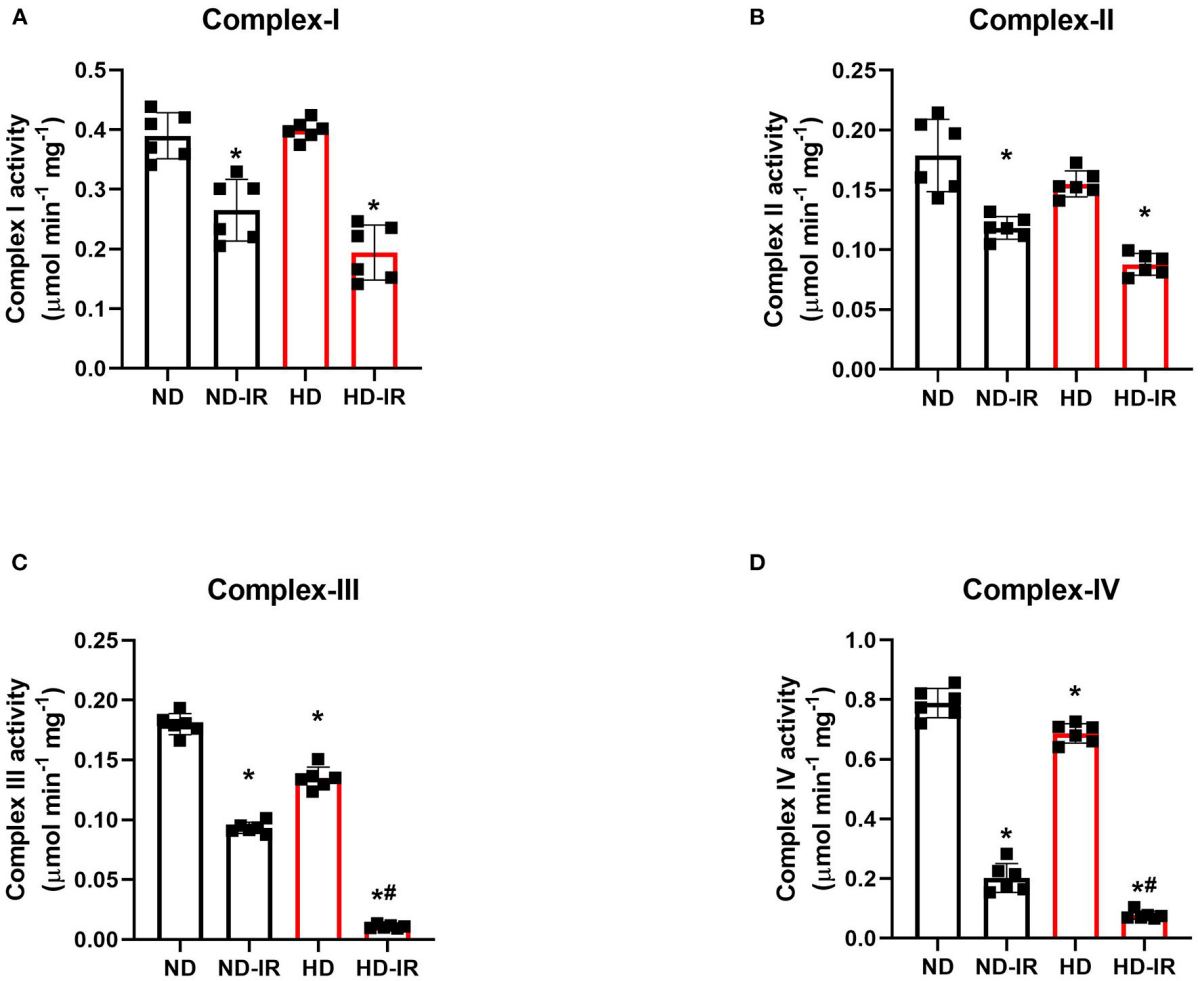
Mitochondrial electron transport chain (ETC) enzymes in ND and HD renal tissue subjected to IR injury. **(A)** Complex I activity, **(B)** Complex II activity, **(C)** Complex III activity, and **(D)** Complex IV activity. Complex I activity was expressed as μmol NADH oxidized/min/mg protein; Complex II activity was expressed in μmol DCPIP reduced/min/mg protein; Complex III activity was expressed in μmol Cytochrome C reduced/min/mg protein; Complex IV activity was expressed in μmol Cytochrome C oxidized/min/mg protein. *p < 0.05 vs. D, ^**#**^*p* < 0.05 vs. ND-IR. The data are presented as mean ± SD (*n* =6/group). ND, Normal diet fed; FD, High fat diet fed; IR, Ischemia-reperfusion.

**Figure 4:**
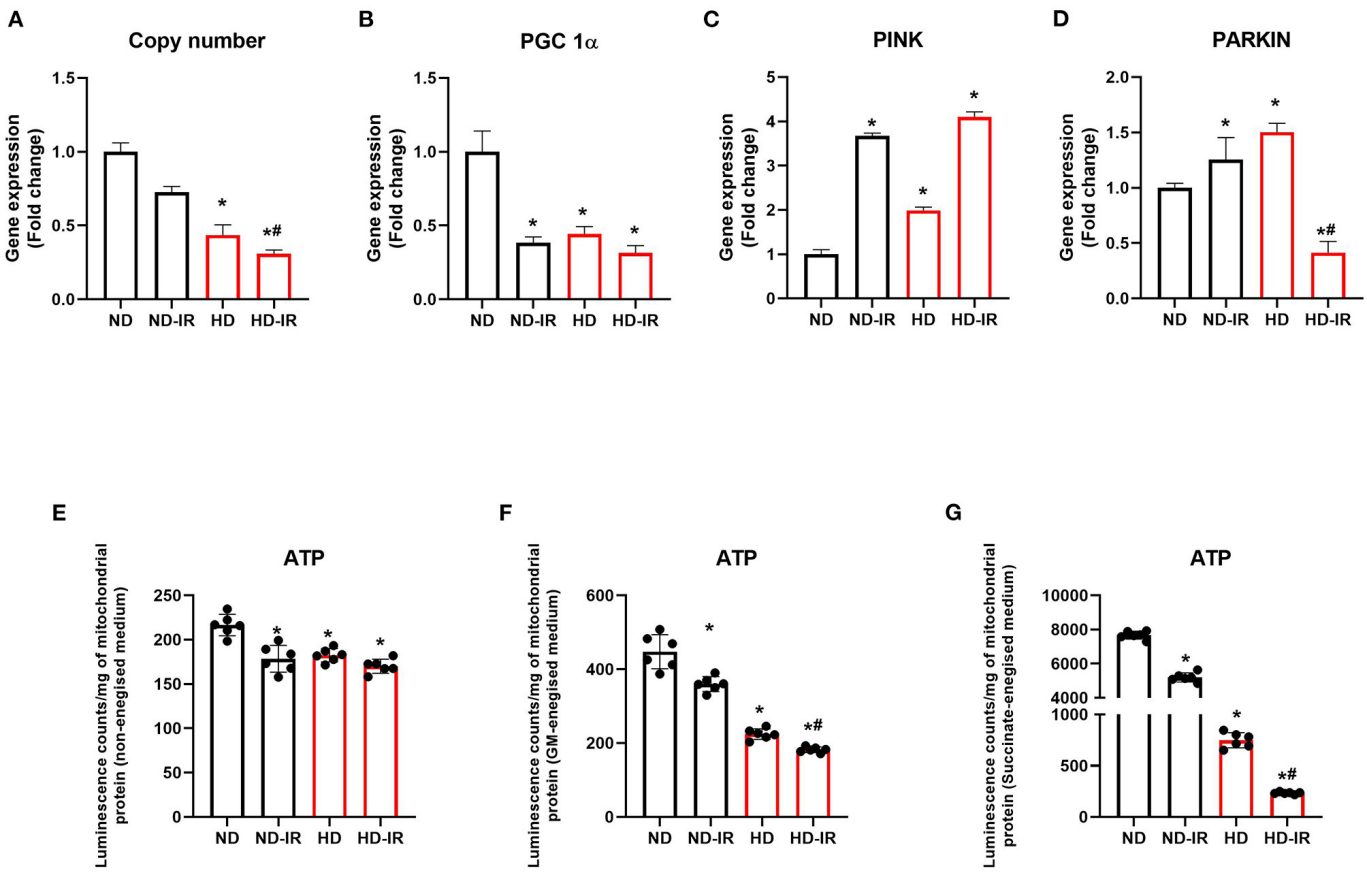
Mitochondrial ATP content and gene expression analysis in ND and HD renal tissue subjected to IR injury. Graph represents **(A)** mitochondrial copy number, gene expression levels of **(B)** PGC-1α, **(C)** PINK, and **(D)** PARKIN in renal tissues. **(E,F)** represents mitochondrial ATP content using ATPlite luminescence kit, in **(E)** non-energized, **(F)** glutamate/malate energized, and **(G)** succinate energized medium. *p < 0.05 vs. ND, ^**#**^*p* < 0.05 vs. ND-IR. The data are presented as mean ± SD (*n* = 6/group). ND, Normal diet fed; FD, High fat diet fed; IR, Ischemia-reperfusion.

Figure 3 shows the impact of IR on the functional activity of mitochondria isolated from the renal tissues conditioned with the different diets. ND and HD rats showed no significant difference in the basal level of proximal ETC complexes (complex I and complex II). In contrast, the distal complex enzymes (Complex III and Complex IV) showed a basal level decline in HD rats (Complex III: ND- 0.17 ± 0.01, HD-0.0.13 ± 0.002; Complex IV: ND- 0.79 ± 0.04, HD-0.0.68 ± 0.02). When subjected to IR, all the complex activities declined in both ND and HD, but there were the prominent decline found in HD (Complex I: ND-IR- 33%, HD-IR- 50%, Complex II- ND-IR- 34%, HD-IR- 51%, Complex III: ND-IR- 47%, HD-IR- 94%, Complex IV: ND-IR- 76%, HD-IR- 90%) (Figures 4A,B). The distal ETC enzyme complexes exhibited a higher deterioration in the activity compared to the proximal complexes.

To assess whether the mitochondrial copy number plays a significant role in the reduced functional activity, we evaluated the mitochondrial copy number in the rat renal tissues from all the experimental groups and noticed a significant (*P* < 0.05) reduction in the copy number in HD treated rats compared to the ND-fed animals (Figure 4A). IR-induced decline in copy number found to be similar in both diet-fed groups from the respective control.

Further analysis of the expression level of PGC-1α (Figure 4B), which plays a critical role in the regulation of mitochondrial biogenesis and respiratory function, showed ≈56% lower expression in the HD control rats than the ND rats. Upon IR, the ND rats showed a decline in the level of PGC 1α, whereas there was no further significant decline observed in HD. To regulate the mitochondrial content and maintain the homeostatic balance, mitophagy plays significant role along with mitochondrial biogenesis. Gene expression analysis of mitophagic genes PINK and PARKIN showed a reduced expression in the HD rats (Figures 4C,D).

We estimated the ATP by using the luminescence kit method to assess whether alterations in the electron transport chain (ETC) affected the ATP production in renal tissue. Figures 4C–E shows the concentration of ATP in mitochondrial fractions of kidneys. At the basal level, ATP content was declined by 16% in HD mitochondria compared to the normal. Upon reperfusion, the level was significantly declined in both ND and HD kidney mitochondrial fractions (ND-IR- 18%, HD-IR- 22%). To assess the IR-induced functional impairment on ATP producing capacity of mitochondria, we incubated the mitochondria in two different energized mitochondrial respiratory mediums, namely, (i) glutamate -malate (GM) and (ii) succinate. After incubating the mitochondria in the respective buffer for 10 min, ATP content was measured by using an ATPlite kit. The mitochondria from ND groups (ND and ND-IR) have retained their ability to generate ATP by utilizing GM or succinate as the substrate. But the HD mitochondrial fraction from sham and IR has lost its ATP-generating capacity considerably in both GM and succinate as substrate. HD-IR showed significant impairment in mitochondrial ATP production compared to ND-IR.

### Effect of High-Fat Diet on the Oxidative Stress Linked to Ischemia-Reperfusion (IR)

The cellular mediators known to be involved in renal IR pathologies are oxidative stress and mitochondrial dysfunction. Accordingly, we measured the oxidative stress in both tissue homogenate and mitochondria in kidney samples (Table 2).

**Table 2:** Oxidative stress analysis and antioxidants levels in the renal homogenate and mitochondria of rats fed with normal diet and high fat diet.

	**ND**			**ND-IR**			**HD**			**HD-IR**		
	**Homogenate**	**Mitochondria**	**Mito/Homo**	**Homogenate**	**Mitochondria**	**Mito/Homo**	**Homogenate**	**Mitochondria**	**Mito/Homo**	**Homogenate**	**Mitochondria**	**Mito/Homo**
Catalase (ml U/mg protein)	15.01± 1.1	3.31 ± 0.1	0.22 ± 0.032	6.881 ± 0.9^*^	1.10 ± 0.14^*^	0.16 ± 0.059^*^	14.36 ± 1.5	3.11 ± 0.32	0.22 ± 0.06	2.99 ± 0.6^*^	0.56 ± 0.03^*^	0.19 ± 0.07
SOD (U/mg protein)	6.63 ± 0.85	4.09 ± 0.38	0.62 ± 0.19	4.56 ± 0.55	2.32 ± 0.47	0.51 ± 0.19	7.92 ± 0.74	3.18 ± 0.42	0.40 ± 0.12^*^	3.83 ± 0.37^*^^#^	1.03 ± 0.15^*^	0.27 ± 0.06^*^^#^
TBARS (nM/mg tissue)	2.12 ± 0.13	1.12 ± 0.08	0.53 ± 0.09	3.12 ± 0.34^*^	1.96 ± 0.10^*^	0.64 ± 0.15^*^	2.64 ± 0.31	1.87 ± 0.201	0.41 ± 0.17	3.24 ± 0.18^*^^#^	2.27 ± 0.31^*^^#^	0.70 ± 0.19^*^^#^
GSH (μM/mg protein)	1.57 ± 0.08	0.14 ± 0.004	0.09 ± 0.01	0.97 ± 0.06^*^	0.08 ± 0.006^*^	0.08 ± 0.01	1.55 ± 0.09	0.12 ± 0.01	0.08 ± 0.01	0.74 ± 0.02	0.05 ± 0.005^*^^#^	0.08 ± 0.006
GSH/GSSG (Ratio)	0.31 ± 0.026	0.68 ± 0.11	2.17 ± 0.75	0.280 ± 0.01	0.39 ± 0.01^*^	1.40 ± 0.12^*^	0.31 ± 0.021	0.5 ± 0.046	1.59 ± 0.35	0.21 ± 0.028^*^^#^	0.3 ± 0.01^*^^#^	1.45 ± 0.26^*^
GPx (μM GSH/mg protein)	0.88 ± 0.047	0.18 ± 0.008	0.21 ± 0.02	0.66 ± 0.044^*^	0.14 ± 0.006	0.22 ± 0.03	0.82 ± 0.023	0.19 ± 0.01	0.23 ± 0.02	0.48 ± 0.015^*^^#^	0.16 ± 0.002	0.33 ± 0.01

*The data are presented as mean ± SD (n = 6/group). ND, Normal diet fed; FD, High fat diet fed; IR, Ischemia-reperfusion*.

* *p < 0.05 vs. ND*,

# *p < 0.05 vs. HD*.

The oxidative stress experienced by the tissue homogenate from ND and HD rats was similar, with insignificant changes in thiobarbituric acid reactive substances (TBARS) and reduced glutathione/oxidized glutathione (GSH/GSSG) level observed between the groups. The results were supported by antioxidant enzymes, such as SOD, catalase, and GPx. However, rat subjected to IR from both the ND and HD groups exhibited decreased GSH/GSSG level with corresponding declined antioxidant enzyme activities in SOD, catalase, and GPx by 31, 54, and 25%, respectively, in ND and 42, 80, and 45%, respectively, in HD compared to the control groups. The IR challenge also escalated the TBARS levels by 32 and 34%, respectively, in ND and HD from control rats (Table 2).

The oxidative stress experienced by the renal mitochondria in IR rats also showed a similar pattern of changes in both oxidative stress markers and antioxidant enzymes, except the changes were more prominent in HD rats. Analyzing the contributory role of mitochondria in total cellular oxidative stress revealed that the ratio between mitochondria/tissue homogenate showed around 35% increase in TBARS level of IR challenged rat from ND and HD groups from respective control. Similarly, GSH/GSSG ratio was declined by 35% in ND-IR rats and only 8% in HD-IR rats from its respective control in the mitochondrial fraction from tissue homogenate (Table 2).

### Effect of High-Fat Diet on the *in vitro* Protein Translational Capacity of Isolated Mitochondria

**Figure 5:**
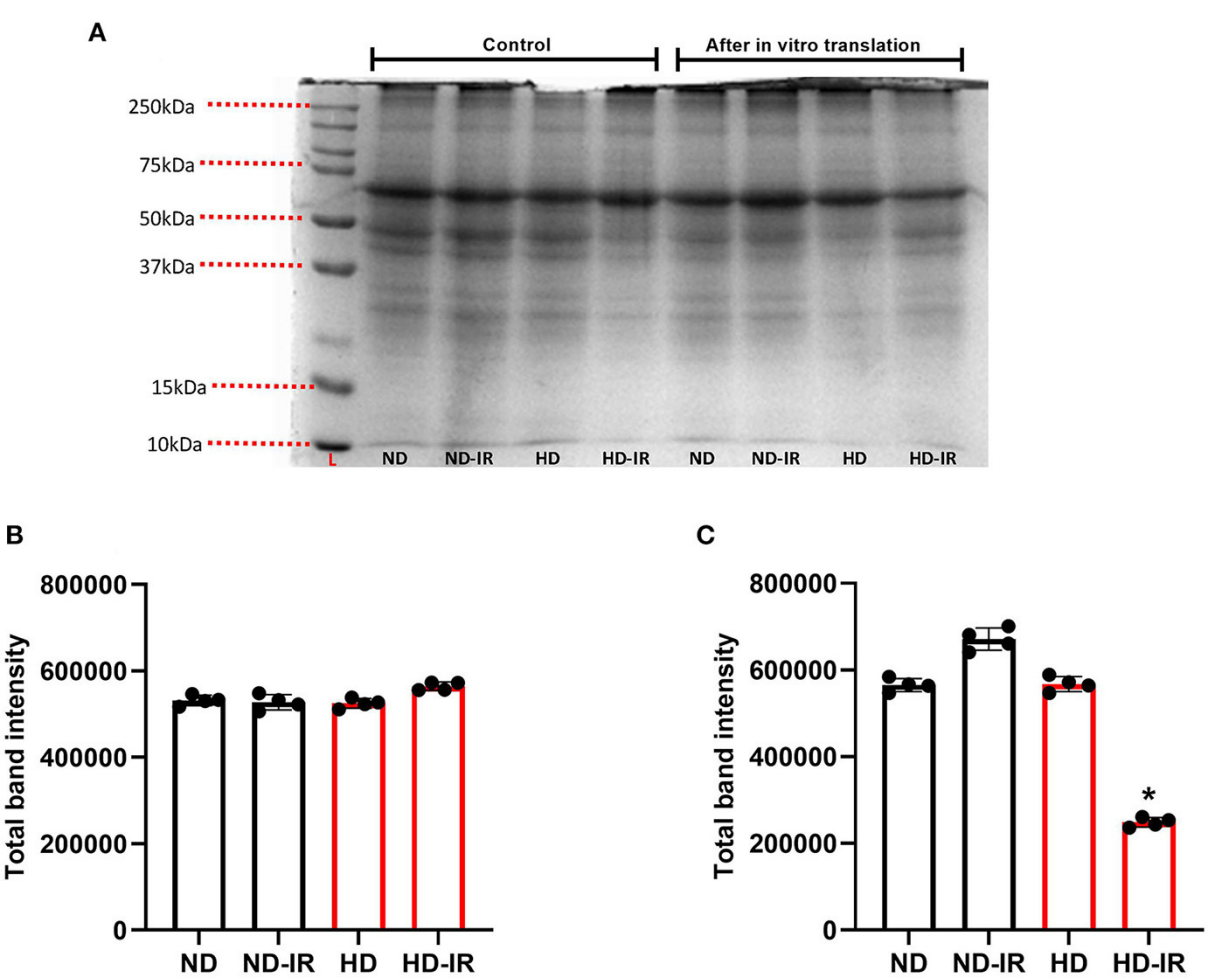
*In-vitro* mitochondrial protein translation analysis in ND and HD renal tissue subjected to IR injury. **(A)** Representative SDS PAGE electrogram, **(B)** total band intensity in the control groups, and **(C)** total band intensity after *in vitro* protein translation. Data were represented as mean ± SD of three individual experiments. *p < 0.05 vs. ND. ND, Normal diet fed; FD, High fat diet fed; IR, Ischemia-reperfusion.

*In vitro* protein translation capacity of isolated mitochondria from different experimental groups was identified and given in Figure 5. Accordingly, the electrogram of protein obtained from SDS PAGE showed no significant difference in the experimental groups except in HD-IR when the mitochondria were incubated in an *in vitro* protein translation buffer without amino acids. However, after the incubation of mitochondria in translation buffer with amino acids, the overall protein level increased in all experimental groups except HD-IR. In fact, the ND-IR group showed increased mitochondrial protein synthesis than its control whereas HD-IR showed a significant decrease from the control.

## Discussion

Recent studies have demonstrated that nutritional interventions can enhance intracellular defense against renal IR (Rysz et al., 2017). However, not much information is available that explains the impact of HD on the outcome of renal IR injury. In this study, we provide evidence for the deleterious effect in rat fed with HD based on the renal function, such as low glomerular filtration rate (GFR), low tissue mitochondrial activities, reduced copy number, and altered mitochondrial gene expression from rats treated with a normal diet. Similarly, when the HD rats were subjected to IR, the eGFR was further declined, corresponding blood renal markers were markedly increased, and renal cell death in HD rats was escalated than ND-IR rats. Similarly, perturbed mitochondrial function was more severe in IR-challenged HD rats than normal diet (ND) rats, which underwent a similar protocol. Further *in vitro* protein translation capacity of isolated mitochondria showed a significant difference between ND and HD rat mitochondria emphasizing the dietary influence on mitochondrial compromised function and response to additional stress.

Kidney-friendly diet can augment the resistance of renal tissue toward IR (Rysz et al., 2017). HD treatment found to exert both protective and deleterious effect according to the organ, for example, the heart (Salie et al., 2014; Webster et al., 2017). This study showed a harmful impact of the HD on kidney. The compromised kidney function and low eGFR exhibited by HD rats in this study indicate altered hemodynamic as there exists a direct relationship between renal blood flow and eGFR. Renal blood flow in turn determinate renal oxygenation where the alterations in the latter can modulate mitochondrial function, increase susceptibility to hypoxia, and disturb the energy demand and balance (Nourbakhsh and Singh, 2014). Accordingly, we found striking mitochondrial functional alterations in HD rats. Experimental induction of IR in HD rat resulted further deteriorated eGFR and indicated the loss of renal tolerance to IR, where renal hypoxia due to hypoperfusion acted as a detrimental factor. Mitochondrial deterioration in IR-challenged HD rats were prominent than ND-IR rats suggests the loss of healthy mitochondria.

Renal tubular injury score measured in the study demonstrated the absence of tubular lesions in HD compared with ND rat kidney. However, glomerular changes, such as glomerular retraction and reduced Bowman's capsular area, were visible in the HD kidney as reported before (Muller et al., 2019). Prominent tubular injury was evident in the samples of IR challenged kidney from both HD and ND, where striking damage was showed in HD-IR rats. The elevated injuries in HD-IR kidney were associated with high mitochondrial oxidative stress and low ETC enzyme activity. Many studies have reported that mitochondrial Complex I and Complex III can act as the sources for ROS release and that the resultant oxidative stress can trigger cellular damage and cell death (Guo et al., 2013; Zorov et al., 2014). Cell death can be initiated by damaged or dysfunctional mitochondria (Green et al., 2011). Through outer membrane permeability pore, mitochondria are involved in both caspase-dependent and caspase-independent cell death (Green et al., 2011). Besides, it can associate with inflammatory response *via* severe damage-associated molecular patterns (DAMPs) (Nakahira et al., 2015).

In addition to IR stimulus, previous studies provide evidence for oxidative stress and mitochondrial dysfunction in HD-fed rat kidney (Sun et al., 2020). Thus, the cumulative impact was the declined mitochondrial copy number in IR-challenged HD rats. Decrease in mitochondrial copy number was reported in 28-week HD-fed rat myocardial tissues in earlier studies (Chen Y. et al., 2018). According to Figure 4, we show a decrease in mitochondrial DNA copy number in HD rats, which suggests impaired mitochondrial bioenergetics. PGC 1α acts as the key regulatory molecule that control mitochondrial biogenesis and metabolism (Gureev et al., 2019). In this study, we found a significant decline in PGC 1α in the renal tissue of HD rats from ND rats. Unlike in ND rats, IR induction did not induce prominent decline in PGC 1α and indicated that the mitochondrial deteriorated function in HD-IR rats was not mainly due to IR induced trigger, rather because of already suppressed basal level. This was evident from the ATP-producing capacity of the mitochondria in different energized medium where the ATP level between HD and HD-IR were not significantly different. Studies have showed that autophagy can determine mitochondrial DNA copy number (Medeiros and Graef, 2019). According to this study, we found that elevated gene expression of PINK and PARKIN in HD kidney from ND indicated possible removal of damaged mitochondria.

To understand the mitochondrial functionality in all experimental groups, we finally assessed the *in vitro* protein translation capacity of isolated mitochondria from different experimental groups. Based on the results given in Figure 5, the total protein separated by SDS PAGE in HD rats subjected to IR was significantly low as compared with the ND rats. Moreover, the pattern of protein bands in the electrogram was also different between ND and HD mitochondria. It indicates that HD treated for 16 weeks modified the renal mitochondrial functional capacity and its ability to respond to cellular stress.

Based on the above observations, we conclude that prolonged HD consumption can adversely affect renal function and its integrity that in turn was rooted in mitochondria. In addition, HD feeding can reduce the tolerance of renal tissue to withstand the ischemic-reperfusion challenge and thereby promote the development of AKI.

## Conclusions

This study shows that high-fat diet (HD) consumption for 16 weeks induced significant changes in rat blood chemistry (where found elevated blood lipid profile and liver injury markers), compromised renal function (reduced eGFR and increased kidney injury markers), and deteriorated mitochondrial function along with low mitochondrial copy number. These diet-induced changes indeed compromised the renal tissue recovery from ischemia reperfusion (IR). The underlying pathology of HD-IR was due to the renal mitochondrial dysfunction.

## Data Availability Statement

The datasets generated and analyzed during the current study are available from the corresponding author on reasonable request.

## Ethics Statement

The animal study was reviewed and approved by Institutional Animal Ethical Committee SASTRA Deemed University, Thirumaisamudram.

## Author Contributions

PP has processed the experimental data, performed analysis, drafted the manuscript, designed figures and tables, and compiled the literature sources. GK has contributed to the design and implementation of the research, to the interpretation of the results, and to the writing of the manuscript. All authors contributed to the article and approved the submitted version.

## Conflict of Interest

The authors declare that the research was conducted in the absence of any commercial or financial relationships that could be construed as a potential conflict of interest.

## Publisher's Note

All claims expressed in this article are solely those of the authors and do not necessarily represent those of their affiliated organizations, or those of the publisher, the editors and the reviewers. Any product that may be evaluated in this article, or claim that may be made by its manufacturer, is not guaranteed or endorsed by the publisher.

## Abbreviations

IR: Ischemia-reperfusion
AKI: Acute kidney injury
CKD: Chronic kidney disease
ND: Normal diet
HD: High-fat diet
eGFR: Estimated glomerular filtration rate
BUN: Blood urea nitrogen
ND-IR: Normal diet-fed rat subjected to ischemia-reperfusion
HD-IR: High fat diet-fed rat subjected to ischemia-reperfusion
K3EDTA: Tripotassium ethylenediaminetetraacetic acid
LDL-C: Low-density lipoprotein cholesterol
HDL-C: High-density lipoprotein cholesterol
SGOT: Serum glutamic-oxaloacetic transaminase
SGPT: serum glutamic-pyruvic transaminase
ETC: Electron transport chain
NADH: Nicotinamide adenine dinucleotide reduced
DCPIP: 2,6-Dichlorophenolindophenol
ATP: Adenosine triphosphate
GSH: Reduced glutathione
GSSG: oxidized glutathione
SOD: Superoxide dismutase
MDA: Malondialdehyde
TBARS: Thiobarbituric acid reactive substances
ND1: NADH-ubiquinone oxidoreductase chain 1
PGC 1α: Proliferator-activated receptor gamma coactivator 1 alpha
UV: Ultraviolet
SDS PAGE: sodium dodecyl sulfate polyacrylamide gel electrophoresis
GM: Glutamate/malate.
